# Understanding disparities in autism diagnosis and care: A socioecological perspective

**DOI:** 10.1371/journal.pmen.0000703

**Published:** 2026-09-10

**Authors:** Abishek Bala, Christa Deban, Henry Hoskinson, Manal Mukhtar, Aditya Shah, Aymen Syeda, Aateqa Hashmi

**Affiliations:** 1 Department of Child and Adolescent Psychiatry, Children’s Hospital of Eastern Ontario, University of Ottawa, Ottawa, Ontario, Canada; 2 Covenant HealthCare College of Medicine at Central Michigan University, Saginaw, Michigan, United States of America; 3 Department of Psychiatry, Covenant HealthCare College of Medicine at Central Michigan University, Saginaw, Michigan, United States of America; PLOS: Public Library of Science, UNITED KINGDOM OF GREAT BRITAIN AND NORTHERN IRELAND

## Abstract

Disparities in autism spectrum disorder (ASD) identification and care are well documented across racial and ethnic, socioeconomic, and geographic lines, yet their underlying mechanisms are incompletely understood. This paper applies the Socioecological Model (SEM) as an organizing framework to examine how individual, interpersonal, community, organizational, and policy-level factors interact to produce inequitable trajectories in ASD diagnosis and service access. At the individual level, heterogeneity in symptom presentation, co-occurring psychiatric conditions, and diagnostic tools normed on Western, predominantly White samples contribute to variation in recognition. Girls from minoritized racial and ethnic backgrounds face compounded delays related to both sex-related camouflaging and cultural assessment bias. At the interpersonal level, provider-family communication, trust, and stigma shape whether developmental concerns are raised, referrals are completed, and services are engaged. Systematic reviews document that caregivers from minoritized groups report lower perceived quality of care and greater barriers to diagnostic follow-through. At the community level, neighborhood disadvantage, school resource variability, and culturally embedded interpretations of child development influence help-seeking and access to early intervention. At the organizational level, inconsistent screening adherence, limited diagnostic capacity, lengthy waitlists, and inequitable reimbursement structures create structural bottlenecks that disproportionately affect publicly insured and minoritized families. At the policy level, variation in state insurance mandates, Medicaid reimbursement rates, workforce distribution, and telehealth regulatory frameworks establish the conditions under which all lower-level factors operate. Recent surveillance data indicate that ASD prevalence among Hispanic, Black, and Asian or Pacific Islander children now exceeds that of White children in some cohorts, reflecting expanded screening and policy reform. However, convergence in prevalence does not constitute equity. Disparities in age at diagnosis, co-occurring intellectual disability, service intensity, and long-term outcomes persist. This paper also addresses training and workforce implications, arguing that sustainable equity requires structural competency education, workforce diversification, and community-partnered approaches integrated across career stages. The SEM framework highlights that disparities in ASD care are not attributable to individual clinical factors alone but emerge from the interaction of relational, institutional, and structural forces. Coordinated, multilevel strategies that extend beyond improved symptom recognition to systemic transformation are necessary to ensure timely, culturally responsive, and universally accessible care for all children.

## Introduction

Autism Spectrum Disorder (ASD) is a prevalent neurodevelopmental condition with a substantial global impact. A recent systematic review estimates global prevalence at approximately 1 in 100 children, with variation across regions and surveillance methodologies [1]. In 2020, one in 36 eight-year-old children in the US met criteria for ASD as reported by the CDC. ASD is characterized by persistent differences in social communication and interaction, alongside restricted and repetitive patterns of behavior, interests, or activities, with onset in early development and clinically significant functional impairment. The heterogeneity of symptom presentation across language, cognitive ability, adaptive functioning, and behavioral regulation contributes to diagnostic complexity.

Epidemiologic surveillance and increased public awareness have contributed to rising identification rates over time. Data from the Global Burden of Disease Study 2021 indicate that ASD remains a leading cause of non-fatal health burden among children and adolescents worldwide [2]. Although early intervention has been shown to improve adaptive functioning and core autism-related outcomes [3], timely access to diagnostic evaluation and evidence-based services remains unevenly distributed.

Racial and ethnic disparities persist in ASD identification and service utilization. Minoritized children often experience delayed diagnosis, differences in service type and intensity, and barriers to specialty care [4]. For example, Medicaid-enrolled White children have been shown to utilize outpatient autism-related services at higher rates than many other racial and ethnic groups, while Black children more frequently receive school-based services [5]. Additionally, emerging work highlights the intersection of assigned sex at birth, race, and ethnicity in shaping age at diagnosis, with compounded delays observed in certain subgroups, including girls from minoritized racial and ethnic backgrounds [6].

These disparities reflect not differences in underlying biology, but inequities embedded within healthcare, educational, and social systems. To understand these multilevel influences, we apply the SEM as a framework for examining how individual, interpersonal, organizational, community, and policy-level factors interact to shape inequitable trajectories in ASD identification and care. This framework allows for a comprehensive review of disparities while also identifying actionable leverage points to promote equity.

## The socioecological model framework

The global health burden of ASD can be quantified through disability-adjusted life years (DALYs), underscoring its substantial and sustained impact across childhood and adolescence [2]. However, this burden is not evenly distributed. Rather, it is shaped by interacting with determinants across multiple ecological levels.

At the individual level, ASD presents with biologically influenced yet highly heterogeneous features. Variability in language development, social reciprocity, and behavioral expression can complicate recognition, particularly in populations whose symptom presentation may differ from traditional diagnostic prototypes. Evidence suggests that diagnostic timing varies according to race, ethnicity, and assigned sex at birth, highlighting the role of bias and structural inequities in clinical interpretation [6].

At the interpersonal level, family knowledge, beliefs, and prior experiences with healthcare systems influence help-seeking behaviors and follow-through on referrals. Trust in providers and effective communication are critical for shared decision-making and engagement in early intervention. However, families from racial and ethnic minority backgrounds report lower-quality encounters and greater barriers to care navigation [4].

At the organizational level, healthcare and educational systems shape referral pathways, service availability, and resource distribution. Disparities in outpatient service use and specialty access among Medicaid-enrolled children demonstrate how insurance structures and institutional processes influence care patterns [5].

At the community level, neighborhood resources, school capacity, and local provider availability contribute to variability in access and timing of diagnosis. Surveillance data show persistent geographic variation in prevalence and age of identification, reflecting differences in infrastructure and service systems rather than true epidemiologic variation [1,2].

At the policy level, screening guidelines, reimbursement structures, workforce distribution, and broader structural inequities determine whether children receive timely evaluation and intervention. Although early intervention improves outcomes [3], policy and financing mechanisms ultimately influence whether families can access those services.

Together, the SEM highlights that inequities in ASD diagnosis and care are not attributable to individual-level factors alone. Instead, they emerge from interacting with structural, institutional, and social forces. Addressing these disparities therefore requires coordinated, multilevel strategies that extend beyond clinical recognition to systemic transformation.

## Individual and family-level factors

At the individual level, disparities in ASD identification are influenced by the heterogeneity of neurodevelopmental presentation. The DSM-5-TR explicitly acknowledges variability in cognitive ability, language development, adaptive functioning, and behavioral expression across the autism spectrum [7]. This heterogeneity complicates early recognition, particularly when symptom presentation diverges from historically prototypical patterns described in predominantly Western samples [8,9]. Variability in adaptive functioning and social communication profiles may obscure diagnostic clarity, especially in children whose presentation does not conform to narrow behavioral expectations.

Sex- and gender-related differences in behavioral expression further influence recognition. Emerging evidence suggests that autistic girls may demonstrate compensatory social strategies, including more reciprocal conversational behaviors and better integration of verbal and nonverbal communication, despite similar underlying social cognition differences compared to boys [10,11]. Social camouflaging or the masking of autistic behaviors through imitation and behavioral adaptation is more frequently reported in females and is associated with delayed diagnosis and increased internalizing symptoms [12]. Co-occurring psychiatric conditions are highly prevalent, with approximately 70% of autistic individuals meeting criteria for at least one comorbid mental disorder [9]. Attention-deficit/hyperactivity disorder, anxiety disorders, and depression are particularly common [13]. These co-occurring conditions may overshadow autism-related features or redirect clinical attention toward alternate diagnoses, thereby contributing to diagnostic delay.

These factors compound across identities. Girls from minoritized racial and ethnic backgrounds may face both sex-related diagnostic delay and cultural assessment bias. Culturally and linguistically diverse (CALD) status has been associated with later age of concern and higher autism severity scores at diagnosis, suggesting intersecting structural and interpretive barriers [14].

Diagnostic criteria and screening tools were largely developed and normed within Western, predominantly Caucasian populations [7]. Cultural differences in norms for social interaction, nonverbal communication, and child behavior influence both symptom expression and clinical interpretation [15]. For example, behaviors such as reduced eye contact or circumscribed interests may carry different cultural meanings, yet standardized tools heavily weight these features without contextual adaptation [16]. Evidence indicates that African American children are more frequently misdiagnosed with adjustment or conduct-related disorders before receiving an autism diagnosis [17]. Furthermore, systematic review data demonstrate that screening instruments developed specifically for the intended cultural population consistently outperform translated Western tools in accuracy and acceptability [18].

Family-level factors, including socioeconomic position, cultural beliefs, and prior experiences with healthcare systems, also shape the trajectory toward diagnosis. Children from lower-income families experience delayed diagnosis and reduced service utilization compared to peers with comparable clinical profiles, suggesting that structural access barriers—rather than intrinsic symptom differences—drive inequitable care trajectories [8]. Although co-occurring psychiatric conditions are common in ASD and may complicate recognition [13], disparities in timing and service engagement persist even when clinical complexity is comparable [8,9].

Cultural beliefs further shape interpretation of developmental differences. Among immigrant Mexican families, autism-related concerns may be understood through frameworks emphasizing relational harmony, family responsibility, and moral interpretations of behavior, sometimes complicating early identification [19]. Similarly, Hispanic immigrant mothers have described interpreting developmental delays through culturally grounded explanatory models before engaging medical systems, often navigating feelings of guilt, fear, and social isolation [20]. Within Latino communities, limited familiarity with the term “autism” and alternative conceptualizations of child development have been associated with delayed diagnosis [21].

Socioeconomic context also influences identification patterns. Children residing in lower socioeconomic neighborhoods are less likely to have documented ASD diagnoses, raising concerns about underidentification in resource-constrained communities [22]. Language environment further intersects with diagnostic disparities; children from non-English-speaking homes have demonstrated lower reported prevalence in school-based surveillance data, suggesting potential barriers in recognition, referral, or classification processes [23]. These patterns likely reflect systemic access differences rather than true variation in incidence [17].

Neighborhood-level and racial/ethnic variation in prevalence estimates underscore the importance of contextual influences. Analyses of county-level trends have demonstrated differential patterns by race/ethnicity and socioeconomic status, suggesting that diagnostic access and service infrastructure contribute significantly to observed disparities [24].

Taken together, while ASD is biologically rooted, individual-level heterogeneity, family context, and cultural interpretation interact with diagnostic frameworks and comorbidity patterns to influence whether and when children are identified. Recognition is therefore shaped not only by symptom expression but also by the lenses through which those symptoms are evaluated.

## Interpersonal-level factors

At the interpersonal level, disparities in ASD identification often emerge within the relational space between caregivers and providers. Even when developmental concerns are present, the quality of communication, trust, and shared decision-making significantly influences whether referrals are made, completed, and followed by engagement in recommended services [17,25].

Caregivers from racially and ethnically minoritized backgrounds frequently report feeling dismissed or not taken seriously when raising developmental concerns. Black caregivers, for example, have described the need to “keep pushing” to obtain referrals for autism evaluation, reflecting perceived minimization of concerns during primary care encounters [26–28]. Such experiences may delay diagnostic ascertainment and erode trust in healthcare systems. Systematic reviews further document that families from minoritized groups report lower perceived quality of care, including reduced shared decision-making and limited cultural sensitivity [25,26].

Autism-related stigma also operates at the interpersonal level. Stigma can influence how families interpret developmental differences and whether they pursue formal evaluation, particularly when diagnostic labeling carries fears of discrimination or social exclusion [29–31]. Among immigrant Mexican families and other Latino communities, autism-related concerns may be understood within culturally grounded explanatory models that shape help-seeking trajectories [21,32,33]. These frameworks do not reflect deficits in knowledge but rather differences in meaning-making that may not align with biomedical diagnostic language.

Provider communication patterns further shape diagnostic pathways. Analyses of clinical documentation and provider practices reveal that implicit bias can influence how concerns are framed and interpreted, with caregivers of color reporting that providers make assumptions about them and delay referrals for evaluation [26,34,35]. When provider language lacks cultural humility or fails to acknowledge family perspectives, it may reinforce disengagement or delay follow-through with services. Conversely, caregivers consistently report valuing respectful communication, collaborative dialogue, and acknowledgment of cultural context [28,32]. The concept of systemic trustworthiness—the extent to which healthcare systems demonstrate reliability, transparency, and responsiveness to the communities they serve—is central to these dynamics. Trust is not simply a characteristic of families but is earned through consistent, respectful, and equitable interactions [27].

Interventions targeting these relational barriers have demonstrated measurable impact. Family navigation models designed to address logistical, psychological, and structural obstacles have significantly improved diagnostic completion among children at risk for ASD, with particularly strong effects among minoritized families [36]. In a randomized clinical trial, children who received family navigation had a greater likelihood of reaching diagnostic ascertainment over one year compared to conventional care management (85.7% vs. 76.4%; unadjusted HR, 1.39; 95% CI, 1.05--1.84) [36]. These findings underscore that when interpersonal processes are strengthened through culturally responsive communication, advocacy, and barrier reduction, inequities in diagnostic follow-through can be mitigated.

Taken together, interpersonal dynamics serve as a critical juncture where structural inequities are either reinforced or interrupted. Disparities in ASD care are therefore not solely a function of symptom presentation but are shaped by relational processes that determine whether families feel heard, supported, and empowered to navigate complex systems [17,26].

## Community-level factors

Community norms and culturally embedded interpretations of child development shape when and how families seek evaluation for ASD. Collective beliefs about language development, temperament, disability, and stigma influence whether developmental differences are normalized, minimized, or medicalized. In Latino communities, limited ASD knowledge, language barriers, diagnostic stress, and mistrust of healthcare systems have been identified as barriers to timely help-seeking [17,33]. Similarly, caregivers from minoritized communities report fear of judgment and community pressure to delay professional evaluation, particularly when diagnostic labeling carries social consequences [26,37]. Black caregivers describe concerns about being labeled as “bad parents” when raising developmental concerns, with these experiences compounded by broader social and systemic mistrust [27]. Among South Asian immigrant families, pronounced autism stigma has been shown to impede timely diagnosis, limit service engagement, and discourage health-promoting behaviors [31]. These findings highlight how community-level stigma and shared norms can delay entry into formal diagnostic systems even before clinical encounters occur.

Community-based interventions demonstrate that these contextual barriers are modifiable. Cultural brokers and trusted community members helped families navigate complex healthcare systems and counter stigma through sustained engagement. Similarly, multi-stage screening protocols embedded within Early Intervention (EI) settings have shown promise in reducing disparities, with no significant differences in participation or referral rates by race, ethnicity, or primary language at any stage of screening [38,39]. Parent-to-parent networks and advocacy organizations further facilitate engagement by providing culturally resonant support, shared lived experience, and practical navigation strategies [26,27]. Community health workers (CHWs) and promotores de salud serve as critical bridges between healthcare systems and communities, providing culturally aligned education, navigation, and support that can reduce mistrust and improve service engagement [33]. Together, these approaches underscore the importance of trusted community infrastructure in mitigating inequities.

Geographic and neighborhood-level inequities also substantially shape access. Children residing in socioeconomically disadvantaged neighborhoods have lower participation rates in EI programs (Part C of the Individuals with Disabilities Education Act [IDEA]) even after receiving an ASD diagnosis. In a population-based study, non-Hispanic Black children had significantly lower odds of EI participation (AOR, 0.67; 95% CI, 0.54--0.84) compared with White peers, while children living in more affluent areas had higher odds of service receipt (AOR, 1.71; 95% CI, 1.36--2.15) [40]. Additional research links neighborhood disadvantage to increased barriers in healthcare utilization despite insurance coverage [41]. Educational systems similarly reflect structural inequities; disparities in access to specialized, school-based, and acute ASD services persist along racial and socioeconomic lines [25]. Furthermore, IDEA Part B Early Childhood Special Education services, which provide supports for children ages 3–5, also exhibit inequitable access, with children from minoritized and lower-income families less likely to receive appropriate services once in school [42]. The intersection of rurality and urbanicity further complicates access; rural communities face profound workforce shortages, limited specialty care, and greater travel burdens, resulting in delayed diagnosis and reduced service intensity [43]. Thus, geography functions as a structural determinant of access, influencing not only whether children are identified but also the intensity and continuity of services received.

Recent surveillance data indicate that ASD prevalence among Hispanic, Black, and Asian/Pacific Islander children has surpassed that of White children in recent birth cohorts [44,45], likely reflecting expanded screening, improved outreach, and policy reforms. However, increased prevalence parity does not equate to equity. Disparities in age at diagnosis, service intensity, and co-occurring intellectual disability remain pronounced. Black and Hispanic children continue to experience later identification relative to White and Asian/Pacific Islander peers [44,46], and Black children with ASD remain more likely to present with co-occurring intellectual disability at diagnosis [47]. These patterns suggest that although detection has improved, inequities in diagnostic timing and service pathways persist.

Collectively, community-level stigma, neighborhood disadvantage, school resource variability, and local service infrastructure continue to produce uneven care trajectories. While awareness and screening reforms have narrowed some gaps in identification, durable equity requires sustained investment in community-based systems that address geographic, cultural, and socioeconomic determinants of access [17,40,41].

## Organizational level factors

At the organizational level, disparities in ASD identification and service access are shaped by institutional workflows, referral systems, workforce distribution, reimbursement participation, and resource allocation within healthcare and educational systems. Even when families actively seek evaluation, structural bottlenecks and inefficient processes can delay diagnostic completion and restrict access to recommended services [48–50]. Thus, inequities often arise not from lack of concern but from how institutions operationalize care delivery.

Implementation of autism screening and referral guidelines varies substantially across healthcare settings. The American Academy of Pediatrics (AAP) recommends autism-specific screening at 18 and 24 months, yet adherence to these guidelines is inconsistent. In pediatric primary care, inconsistent adherence to developmental surveillance and referral protocols contributes to missed or delayed identification, particularly among children from minoritized backgrounds. In one large primary care network, only 40.2% of children who screened positive received at least one recommended referral, and just 3.7% received all recommended referrals; disparities were observed by sex, language, socioeconomic status, and race [35]. Screening completion, referral tracking, and care coordination frequently depend on clinic-level infrastructure, staffing, and electronic health record integration. In under-resourced systems, competing demands and limited specialty capacity extend the interval between concern and formal evaluation [26,34]. The decentralized and siloed nature of healthcare, educational, and social service systems further exacerbates these delays, as families must navigate disconnected organizations with limited coordination.

Diagnostic capacity itself is unevenly distributed. Comprehensive multidisciplinary evaluations often involve lengthy waitlists, commonly exceeding 6–12 months—particularly within safety-net systems and rural or underserved regions [48,51]. Workforce shortages in developmental-behavioral pediatrics (DBP), child psychology, speech-language pathology, and child psychiatry exacerbate these delays [43]. Traditional multidisciplinary models, while clinically robust, are resource-intensive and costly to insurers, inconvenient for families, and disproportionately burdensome for those with limited flexibility or transportation [51]. Organizational triage systems may prioritize more overt behavioral presentations, inadvertently disadvantaging children with less externalizing symptom profiles.

Insurance participation and reimbursement structures further influence access at the organizational level. Specialty clinics may limit Medicaid acceptance due to lower reimbursement rates, concentrating publicly insured families into fewer diagnostic sites and extending wait times. Higher state Medicaid reimbursement rates have been associated with lower odds of access-related care problems for children with autism [52]. Administrative barriers including prior authorizations, documentation requirements, and rigid eligibility criteria introduce friction that disproportionately affects families with fewer logistical resources [34]. Conversely, Medicaid Home and Community-Based Services waivers with higher cost and enrollment limits have been associated with reductions in unmet healthcare needs among children with ASD [53], illustrating how organizational-level financing structures shape service uptake. Insurance type—including dual enrollment in Medicaid and private insurance—can also affect access, as families with dual coverage may face different provider networks and authorization requirements [54].

Educational systems reflect parallel organizational inequities. Schools often serve as primary referral pathways for evaluation, yet disparities in staffing ratios, access to school psychologists, and availability of autism-specific resources influence identification and service intensity. Under-resourced districts may lack capacity for comprehensive evaluation, contributing to delayed Individualized Education Program (IEP) development and inconsistent service provision [17,42]. Disproportionality in autism special education eligibility may reflect inequitable evaluation processes, including less comprehensive assessments and reduced access to autism-specific measures for minoritized students [42]. Variability in interpretation of eligibility criteria across districts further compounds disparities.

Data infrastructure within organizations also plays a critical role. Many healthcare and educational systems lack routine monitoring of diagnostic timelines, referral completion, and service utilization stratified by race, ethnicity, language, or neighborhood. Without systematic disparity tracking, inequities remain embedded within institutional processes and are less likely to be addressed through quality improvement initiatives [34].

Importantly, organizational systems can be redesigned to interrupt inequity. Integrated care models, tiered diagnostic pathways, and embedded primary care evaluations have demonstrated substantial reductions in wait times and improvements in access. A fast-track triage model in an integrated community specialty clinic achieved a median age of diagnosis of 33 months compared to national and state medians of 49 and 59 months, respectively [55]. The (S)TAAR transdisciplinary model increased patient throughput, reduced waitlists, and achieved equitable improvements across race, ethnicity, language, and insurance type [56]. Primary care-embedded autism evaluation programs have demonstrated significantly shorter wait times while serving predominantly Black and Latinx children insured by Medicaid [57]. Similarly, a statewide tiered screening and diagnostic system in Indiana evaluated more than 2,000 children with a median wait time of 62 days [58]. Streamlined referral processes, structured navigation supports, and standardized follow-up protocols consistently narrow disparities in diagnostic completion [59,60].

Organizational systems therefore function as pivotal intermediaries between community determinants and broader policy structures. Institutional workflows, workforce capacity, reimbursement participation, and accountability mechanisms collectively determine whether children move efficiently from developmental concern to diagnostic clarity and sustained intervention. Advancing equity in ASD care requires deliberate organizational redesign alongside broader structural reform [17,48].

## Policy and structural factors

At the policy and structural level, disparities in ASD diagnosis and service access are shaped by financing systems, legislative mandates, workforce investment, and regulatory frameworks that define how care is funded, incentivized, and distributed. Unlike organizational barriers, which reflect how institutions implement care, structural determinants establish the resource environment within which those institutions operate [54,61]. These upstream forces influence provider availability, insurance participation, service intensity, and ultimately, diagnostic timing.

Insurance policy is a central structural determinant of access. Although all U.S. states mandate some level of autism insurance coverage, substantial variation persists in reimbursement rates, eligibility criteria, annual caps, and enforcement mechanisms [54,61]. State mandates have demonstrably expanded access under commercial insurance; implementation was associated with a 3.4-percentage-point increase in monthly service use and a $77 increase in monthly ASD-related spending [62]. Treated prevalence of ASD increased approximately 10% following mandate implementation, rising to 18% several years post-enactment [63]. However, structural inequities remain pronounced in publicly insured populations. Lower Medicaid reimbursement rates are associated with reduced provider participation and greater access-related challenges, whereas higher reimbursement rates correlate with fewer reported barriers [52]. State-level differences in Medicaid Home and Community-Based Services waiver caps and enrollment limits further shape service intensity; more generous waivers are associated with decreased unmet healthcare needs among children with ASD [53]. Eligibility criteria for autism-related services, such as the presence of co-occurring intellectual disability, can also restrict access [54]. Thus, financing policies produce uneven access landscapes across states and demographic groups.

Workforce policy represents another critical structural lever. National shortages in DBP, child psychiatry, psychology, and speech-language pathology disproportionately affect rural and historically marginalized communities [64,43]. In 2023, there were only 1.0 DBP subspecialists per 100,000 U.S. children, with substantial geographic variability; projections suggest minimal workforce expansion through 2040 [64]. The DBP workforce reports high complexity, extended waitlists, significant nonreimbursed workload, and anticipated retirements, further constraining access [65]. Broader autism-related workforce availability remains limited in total numbers, time capacity, and specialty expertise, with the greatest unmet need among racial and ethnic minorities and rural populations [43]. While insurance mandates were associated with a 16% increase in board-certified behavior analysts between 2003 and 2017, effects on child psychiatrists and pediatricians were limited [66], suggesting that targeted workforce development strategies are necessary to address specialty shortages and distribution inequities.

Regulatory frameworks further shape equitable access. Multidisciplinary diagnostic requirements—though clinically rigorous—can exacerbate bottlenecks in settings with limited workforce supply [51]. Telehealth policy expansion during the COVID-19 pandemic demonstrated potential to mitigate geographic barriers; tele-assessment for ASD has shown 80--91% diagnostic concordance with in-person evaluations, with clinicians able to make determinations for the majority of children assessed [58,67]. However, variability in reimbursement parity and cross-state licensure regulations limits sustainability of telehealth-based diagnostic models. Providers and caregivers have raised concerns regarding telehealth equity, particularly for families with limited broadband access or preferred languages other than English [68]. Policy decisions regarding telehealth reimbursement and licensure portability therefore carry direct implications for equitable reach.

Federal and state accountability mechanisms influence surveillance and investment. The Autism and Developmental Disabilities Monitoring (ADDM) Network has documented substantial increases in ASD prevalence and shifting racial and ethnic patterns in identification. In 2020, one in 36 eight-year-old children met criteria for ASD, and for the first time, prevalence was lower among White children than among several minoritized groups [44]. Yet improved detection does not eliminate inequity. Disparities in age at diagnosis and co-occurring intellectual disability persist; Black children with ASD remain more likely than White children to have intellectual disability (50.8% vs. 31.8%), and Black children with intellectual disability are diagnosed at later median ages [44,69]. Additionally, neighborhood-level social vulnerability interacts with race and ethnicity in complex ways, with identification patterns differing across communities of varying vulnerability [70]. These findings suggest that structural context continues to shape not only whether children are identified, but how early and with what accompanying service trajectories.

Importantly, convergence in prevalence does not equate to equity. While identification by 48 months was four times more likely in 2016 than in 2002, and gains were particularly pronounced among children without intellectual disability, substantial racial and ethnic disparities in early identification remain [46]. Increases in prevalence among Hispanic, Black, and Asian/Pacific Islander children likely reflect expanded screening and policy reform rather than true epidemiologic shifts [44,71]. Structural inequities continue to manifest in diagnostic timing, service intensity, and long-term developmental outcomes.

Policy and structural determinants therefore define the outer architecture of ASD care systems. Financing formulas, workforce investment, regulatory flexibility, and accountability mechanisms establish the conditions under which organizations operate and families navigate services. Sustainable equity in ASD care requires deliberate structural reform—aligning reimbursement, workforce development, telehealth regulation, and accountability systems with the goal of universal, timely, and culturally responsive access [61,43].

## Training and educational implications

Reducing inequities in ASD identification and service access requires coordinated workforce development across disciplines and career stages. Training must move beyond episodic “cultural competence” modules toward longitudinal, competency-based preparation that integrates structural drivers of disparity, culturally responsive assessment, and community-engaged care as core clinical skills [17,15,72].

## Structural competency as foundational training

Pre-service education for psychologists, educators, physicians, social workers, and allied health professionals should embed equity as a longitudinal thread throughout didactics, supervision, and evaluation. Trainees must develop expertise not only in diagnostic criteria and psychometric tools, but also in understanding how race, ethnicity, language, immigration context, socioeconomic position, and disability intersect to shape developmental trajectories and care pathways [16,73].

Structural competency offers a practical framework for this integration. Metzl and Hansen (2014) describe five core competencies:

Recognizing the structures that shape clinical interactions
Developing an extra-clinical language of structure
Reframing “cultural” formulations in structural terms
Imagining and implementing structural interventions
Practicing structural humility

Residents who participated in structural competency curricula reported improved awareness of how institutional and social forces influence patient outcomes and described meaningful changes in their clinical relationships, though they also identified the need for sustained institutional support to translate awareness into systemic change [74]. This suggests that equity-oriented training must be continuous, practice-integrated, and institutionally reinforced rather than conceptual alone.

## Workforce diversification as an equity intervention

Workforce diversification is itself a structural equity strategy. Recruitment, retention, and advancement of professionals from historically underrepresented backgrounds are associated with improved patient trust, communication quality, and service engagement [75,76]. Racial concordance between patients and clinicians increases visit likelihood, enhances communication effectiveness, and improves patient-reported outcomes [76]. At the population level, greater Black primary care physician representation has been associated with increased life expectancy among Black individuals, particularly in high-poverty counties [77].

Importantly, minority physicians disproportionately serve minority and publicly insured populations. One study found that minority physicians cared for substantially more minority patients (63% vs. 42%) and more uninsured or Medicaid patients (53% vs. 40%) than their non-minority peers [75]. Without deliberate investment in pipeline programs, mentorship structures, equitable promotion pathways, and inclusive institutional climates, workforce expansion alone will not correct maldistribution.

## Practical implementation skills

Equity-focused training must also prioritize actionable clinical competencies. These include culturally responsive interviewing, effective interpreter use, recognition and repair of bias in diagnostic reasoning, and familiarity with community navigation pathways. The ECLECTIC framework provides one example of a structured cultural assessment model that enhances clinician awareness of bias during ASD evaluation and supports culturally relevant recommendations [16].

Evidence increasingly supports the effectiveness of culturally responsive ASD interventions. A recent meta-analysis demonstrated large and significant overall effect sizes for culturally adapted interventions, particularly in social communication, mental health outcomes, and caregiver implementation fidelity [78]. These findings underscore that equity-informed approaches are not merely ethical imperatives—they are clinically effective.

Experiential learning in under-resourced and community-based settings further strengthens readiness. Co-design initiatives with culturally embedded community organizations have identified key features for improving access, including respecting diversity, prioritizing caregiver agency, reducing stigma, and maximizing feasibility [79]. Training models that incorporate community partnership and co-design foster structural humility and practical problem-solving capacity.

## Continuing professional development and accountability

Continuing education for practicing clinicians and educators is equally essential. National assessments indicate that formal training in health disparities remains inconsistent across residency programs [80]. Integrating equity-focused content into continuing medical education, scientific conferences, and professional development initiatives enhances clinician familiarity with social determinants and structural drivers of disease [80].

Longitudinally integrated social justice curricula have demonstrated that students exposed to sustained equity training feel better prepared to serve underserved populations than peers without such exposure [81]. Importantly, continuing education must be linked to institutional accountability—such as tracking diagnostic timelines, referral completion rates, and service utilization stratified by race, ethnicity, language, and insurance status—to ensure that training translates into measurable system improvement.

Workforce preparation serves as the implementation bridge between structural reform and equitable care. Policy can expand coverage and resources, but training determines whether those expanded systems operate justly. Sustainable progress in ASD equity therefore depends on aligning reimbursement structures, workforce development, regulatory frameworks, and educational transformation toward a unified goal: timely, culturally responsive, and universally accessible care [82,83].

## Future directions

Disparities in ASD identification and service access arise from interacting influences across the socioecological spectrum rather than from individual differences alone [17,84]. Variation in symptom presentation and diagnostic complexity may affect recognition, but persistent inequities in age at diagnosis, service use, and outcomes reflect factors beyond the child. These include interpersonal, community, organizational, and structural determinants [25,46]. The SEM highlights how disparities emerge across clinical interactions, community environments, institutional systems, financing structures, workforce distribution, and policy frameworks [34,48].

At the interpersonal level, communication quality, trust, stigma, and provider bias influence whether developmental concerns are recognized and referrals occur [27,35]. Community factors such as neighborhood disadvantage, school resources, and cultural interpretations of development influence help seeking and access to early intervention [40]. Organizational systems shape whether families experience efficient diagnostic pathways or prolonged delays. Structural policies influence reimbursement, workforce capacity, and regulatory conditions that affect service availability [48]. Workforce training plays an important role by translating structural reforms into everyday clinical practice [72].

The role of research itself must also be critically examined. Historically, autistic individuals from racially and socioeconomically marginalized communities have been underrepresented in research, limiting the generalizability of findings and perpetuating inequities [85,86]. Research design and recruitment must be intentionally inclusive and culturally responsive, ensuring that interventions are relevant and effective for the diverse populations they aim to serve [85,87]. Participatory approaches such as community-based participatory research (CBPR) allow community members to participate as partners in study design and implementation, fostering trust and ensuring that research addresses community priorities [88,89]. Diversity advisory boards and culturally responsive recruitment strategies may further support inclusive research participation [90]. Black and African American families have identified culturally aligned research teams, access to information, and opportunities for support as important facilitators of research participation [91].

Future research should prioritize multilevel implementation strategies. Studies should examine how coordinated interventions across SEM levels affect diagnostic timing, service engagement, and long-term outcomes. Combining strategies such as family navigation, workflow redesign, and reimbursement reform may produce greater improvements than isolated interventions. Habayeb et al. (2023) describe a multisystem approach that integrates infrastructure development, enabling services, and direct clinical care to address barriers across levels. Similar models require further evaluation, particularly in historically marginalized and under-resourced communities [84,92].

Improved data infrastructure is also needed. Healthcare and educational systems should routinely track diagnostic timelines, referral completion, service intensity, and outcomes. These measures should be stratified by race, ethnicity, language, insurance status, and neighborhood social vulnerability [47,93]. Equity metrics should be integrated into quality improvement efforts rather than treated as supplemental reporting.

Increasing ASD prevalence across racial and ethnic groups does not necessarily indicate equity. Although ASD prevalence among Hispanic, Black, and Asian or Pacific Islander children now exceeds that of White children in some cohorts, disparities in age at diagnosis, co-occurring intellectual disability, service intensity, and long-term outcomes remain [46,47,93]. Improved detection reflects progress in screening and policy reform but does not eliminate structural inequities.

Reducing ASD disparities requires coordinated change across socioecological levels. Structural investment, organizational redesign, community partnership, workforce development, and inclusive research must work together. Sustainable equity will depend on ensuring that every child, regardless of race, ethnicity, language, geography, or socioeconomic context, has timely access to accurate diagnosis and culturally responsive intervention [17,84]. The SEM provides both an explanation of current disparities and a framework for guiding future action.

## References

[1] Zeidan J, Fombonne E, Scorah J, Ibrahim A, Durkin MS, Saxena S, et al. Global prevalence of autism: a systematic review update. Autism Res. 2022;15(5):778–90. doi: 10.1002/aur.2696 35238171 PMC9310578

[2] Global Burden of Disease Study 2021 Autism Spectrum Collaborators. The global epidemiology and health burden of the autism spectrum: findings from the Global Burden of Disease Study 2021. Lancet Psychiatry. 2025;12(2):111–21. doi: 10.1016/S2215-0366(24)00363-8 39709974 PMC11750762

[3] Estes A, Munson J, Rogers SJ, Greenson J, Winter J, Dawson G. Long-term outcomes of early intervention in 6-year-old children with autism spectrum disorder. J Am Acad Child Adolesc Psychiatry. 2015;54(7):580–7. doi: 10.1016/j.jaac.2015.04.005 26088663 PMC4475272

[4] Brasher S, Stapel-Wax JL, Muirhead L. Racial and ethnic disparities in autism spectrum disorder: implications for care. Nurs Clin North Am. 2022;57(3):489–99. doi: 10.1016/j.cnur.2022.04.014 35985735

[5] Bilaver LA, Sobotka SA, Mandell DS. Understanding racial and ethnic disparities in autism-related service use among medicaid-enrolled children. J Autism Dev Disord. 2021;51(9):3341–55. doi: 10.1007/s10803-020-04797-6 33219917 PMC8137720

[6] Goldblum JE, McFayden TC, Bristol S, Putnam OC, Wylie A, Harrop C. Autism prevalence and the intersectionality of assigned sex at birth, race, and ethnicity on age of diagnosis. J Autism Dev Disord. 2024;54(10):3777–91. doi: 10.1007/s10803-023-06104-5 37584770 PMC10869641

[7] Jeste SS, Geschwind DH. Disentangling the heterogeneity of autism spectrum disorder through genetic findings. Nat Rev Neurol. 2014;10(2):74–81. doi: 10.1038/nrneurol.2013.278 24468882 PMC4125617

[8] Hirota T, King BH. Autism spectrum disorder: a review. JAMA. 2023;329(2):157–68. doi: 10.1001/jama.2022.23661 36625807

[9] Lord C, Elsabbagh M, Baird G, Veenstra-Vanderweele J. Autism spectrum disorder. Lancet. 2018;392(10146):508–20. doi: 10.1016/S0140-6736(18)31129-2 30078460 PMC7398158

[10] Lai M-C, Szatmari P. Sex and gender impacts on the behavioural presentation and recognition of autism. Curr Opin Psychiatry. 2020;33(2):117–23. doi: 10.1097/YCO.0000000000000575 31815760

[11] Hodge MA, Sutherland R, Boulton KA, Baracz SJ, Ong N, Bennett B, et al. Focusing on autism symptoms masks sex-specific needs of autistic children. Autism. 2025;29(5):1318–32. 39704418 10.1177/13623613241303550

[12] McQuaid GA, Lee NR, Wallace GL. Camouflaging in autism spectrum disorder: Examining the roles of sex, gender identity, and diagnostic timing. Autism. 2022;26(2):552–9. doi: 10.1177/13623613211042131 34420418

[13] Kirsch AC, Huebner ARS, Mehta SQ, Howie FR, Weaver AL, Myers SM, et al. Association of comorbid mood and anxiety disorders with autism spectrum disorder. JAMA Pediatr. 2020;174(1):63–70. doi: 10.1001/jamapediatrics.2019.4368 31790555 PMC6902186

[14] Hussain A, John JR, Dissanayake C, Frost G, Girdler S, Karlov L, et al. Sociocultural factors associated with detection of autism among culturally and linguistically diverse communities in Australia. BMC Pediatr. 2023;23(1):415. doi: 10.1186/s12887-023-04236-2 37612588 PMC10463473

[15] de Leeuw A, Happé F, Hoekstra RA. A conceptual framework for understanding the cultural and contextual factors on autism across the globe. Autism Res. 2020;13(7):1029–50. doi: 10.1002/aur.2276 32083402 PMC7614360

[16] Bordes Edgar V, Meneses V, Shaw D, Sotomayor M, Fujii D. Clinical utility of the ECLECTIC framework in providing culturally informed ASD evaluations. Clin Neuropsychol. 2022;36(5):1148–71. 34126862 10.1080/13854046.2021.1936187

[17] Pham AV, Charles LC. Racial disparities in autism diagnosis, assessment, and intervention among minoritized youth: sociocultural issues, factors, and context. Curr Psychiatry Rep. 2023;25(5):201–11. doi: 10.1007/s11920-023-01417-9 37004631

[18] Huda E, Hawker P, Cibralic S, John JR, Hussain A, Diaz AM, et al. Screening tools for autism in culturally and linguistically diverse paediatric populations: a systematic review. BMC Pediatr. 2024;24(1):610. doi: 10.1186/s12887-024-05067-5 39342198 PMC11437884

[19] Cohen SR, Miguel J. Amor and social stigma: ASD beliefs among immigrant Mexican parents. J Autism Dev Disord. 2018;48(6):1995–2009. doi: 10.1007/s10803-017-3457-x 29318433

[20] Ijalba E. Hispanic immigrant mothers of young children with autism spectrum disorders. Am J Speech Lang Pathol. 2016;25(2):200–13. 27135836 10.1044/2015_AJSLP-13-0017

[21] Zuckerman KE, Sinche B, Cobian M, Cervantes M, Mejia A, Becker T, et al. Conceptualization of autism in the Latino community and its relationship with early diagnosis. J Dev Behav Pediatr. 2014;35(8):522–32. doi: 10.1097/DBP.0000000000000091 25186120 PMC4180801

[22] Dickerson AS, Rahbar MH, Pearson DA, Kirby RS, Bakian AV, Bilder DA, et al. Autism spectrum disorder reporting in lower socioeconomic neighborhoods. Autism. 2017;21(4):470–80. doi: 10.1177/1362361316650091 27627912

[23] Dickerson AS, Dickerson AS. Brief report: Texas school district autism prevalence in children from non-English-speaking homes. J Autism Dev Disord. 2020;50(4):1411–7. doi: 10.1007/s10803-018-3676-9 29974313

[24] Nevison C, Parker W. California autism prevalence by county and race/ethnicity: declining trends among wealthy whites. J Autism Dev Disord. 2020;50(11):4011–21. doi: 10.1007/s10803-020-04460-0 32193763 PMC7557477

[25] Smith KA, Gehricke J-G, Iadarola S, Wolfe A, Kuhlthau KA. Disparities in service use among children with autism: a systematic review. Pediatrics. 2020;145(Suppl 1):S35–46. doi: 10.1542/peds.2019-1895G 32238530

[26] Fisher AP, Lynch JD, Jacquez FM, Mitchell MJ, Kamimura-Nishimura KI, Wade SL. A systematic review examining caregivers’ of color experiences with the diagnostic process of autism spectrum disorder. Autism. 2023;27(4):876–89. doi: 10.1177/13623613221128171 36321366

[27] Onovbiona H, Quetsch L, Bradley R. Racial and practical barriers to diagnostic and treatment services for black families of autistic youth: a mixed-method exploration. J Autism Dev Disord. 2024;54(12):4465–80. doi: 10.1007/s10803-023-06166-5 38038872

[28] Weitlauf AS, Miceli A, Vehorn A, Dada Y, Pinnock T, Harris JW, et al. Screening, diagnosis, and intervention for autism: experiences of black and multiracial families seeking care. J Autism Dev Disord. 2024;54(3):931–42. doi: 10.1007/s10803-022-05861-z 36626007 PMC10330934

[29] Kinnear SH, Link BG, Ballan MS, Fischbach RL. Understanding the experience of stigma for parents of children with ASD. J Autism Dev Disord. 2016;46(3):942–53. 26659549 10.1007/s10803-015-2637-9

[30] Turnock A, Langley K, Jones CRG. Understanding stigma in autism: a narrative review and theoretical model. Autism Adulthood. 2022;4(1):76–91. doi: 10.1089/aut.2021.0005 36605561 PMC8992913

[31] Shafi A, Mohd Hussin H, Nor MNM. Autism stigma among South Asian immigrant families and its impact on diagnosis and service engagement. J Autism Dev Disord. 2024.

[32] Coffield CN, Spitalnik DM, Harris JF, Jimenez ME. Exploring the experiences of families of Latino children newly diagnosed with autism spectrum disorder. J Dev Behav Pediatr. 2021;42(9):711–6. doi: 10.1097/DBP.0000000000000965 33941738

[33] Zuckerman KE, Lindly OJ, Reyes NM, Chavez AE, Macias K, Smith KN, et al. Disparities in diagnosis and treatment of autism in Latino and Non-Latino White families. Pediatrics. 2017;139(5):e20163010. doi: 10.1542/peds.2016-3010 28557734 PMC5404727

[34] Srivarathan A, Bradford A, Shearkhani S. Bridging diagnostic safety. BMJ Qual Saf. 2025. 10.1136/bmjqs-2025-018723 PMC12707049 40854799

[35] Wallis KE, Guthrie W, Bennett AE, Gerdes M, Levy SE, Mandell DS, et al. Adherence to screening and referral guidelines for autism spectrum disorder in toddlers in pediatric primary care. PLoS One. 2020;15(5):e0232335. doi: 10.1371/journal.pone.0232335 32379778 PMC7205236

[36] Feinberg E, Augustyn M, Broder-Fingert S, Bennett A, Weitzman C, Kuhn J, et al. Effect of family navigation on diagnostic ascertainment among children at risk for autism: a randomized clinical trial from DBPNet. JAMA Pediatr. 2021;175(3):243–50. doi: 10.1001/jamapediatrics.2020.5218 33427861 PMC7802008

[37] Rivera-Figueroa EI, Ruiz Y, Rosario-Flores M. Fear of judgment and community pressure as barriers to autism diagnosis among Black caregivers. J Autism Dev Disord. 2022.

[38] Eisenhower A, Martinez Pedraza F, Sheldrick RC, Frenette E, Hoch N, Brunt S, et al. Multi-stage screening in early intervention: a critical strategy for improving ASD identification and addressing disparities. J Autism Dev Disord. 2021;51(3):868–83. doi: 10.1007/s10803-020-04429-z 32144605 PMC7483311

[39] Sheldrick RC, Carter AS, Eisenhower A, Mackie TI, Cole MB, Hoch N, et al. Effectiveness of screening in early intervention settings to improve diagnosis of autism and reduce health disparities. JAMA Pediatr. 2022;176(3):262–9. doi: 10.1001/jamapediatrics.2021.5380 34982099 PMC8728657

[40] Shenouda J, Barrett E, Davidow AL, Sidwell K, Halperin W, Silenzio VMB, et al. Disparities in early intervention program participation by children with autism spectrum disorder in a US metropolitan area, 2006 to 2016. JAMA Pediatr. 2022;176(9):906–14. doi: 10.1001/jamapediatrics.2022.2366 35849409 PMC9295023

[41] Yu SY, Neely T, Malow BA. Neighborhood disadvantage and healthcare utilization barriers despite insurance coverage in autism. J Autism Dev Disord. 2024.

[42] Young K, Harris B, Hall-Lande J, Esler A. The intersection of systemic, child, and evaluation factors in autism special education eligibility. J Autism Dev Disord. 2024;54(9):3274–89. 37480439 10.1007/s10803-023-06059-7

[43] McBain RK, Kareddy V, Cantor JH, Stein BD, Yu H. Systematic review: united states workforce for autism-related child healthcare services. J Am Acad Child Adolesc Psychiatry. 2020;59(1):113–39. doi: 10.1016/j.jaac.2019.04.027 31150751 PMC6883168

[44] Maenner MJ, Warren Z, Williams AR. Prevalence and characteristics of ASD among children aged 8 years - ADDM Network, United States, 2020. MMWR Surveill Summ. 2023;72(2):1–14. 10.15585/mmwr.ss7202a1 PMC10042614 36952288

[45] Gallin Z, Kolevzon AM, Reichenberg A, Hankerson SH, Kolevzon A. Racial differences in the prevalence of autism spectrum disorder: a systematic review. J Autism Dev Disord. 2025;55(9):3364–77. doi: 10.1007/s10803-024-06403-5 38941049 PMC12367818

[46] Shaw KA, McArthur D, Hughes MM, et al. Progress and disparities in early identification of ASD: ADDM Network, 2002-2016. J Am Acad Child Adolesc Psychiatry. 2022;61(7):905–14. 34838692 10.1016/j.jaac.2021.11.019 PMC9353949

[47] O’Sharkey K, Mitra S, Paik SA. Trends in ASD prevalence in California. J Autism Dev Disord. 2025;55(7):2503–11. 38700778 10.1007/s10803-024-06371-w PMC12167244

[48] Habayeb S, Inge A, Myrick Y, Hastings A, Long M, Hoffman SB, et al. A multisystem approach to improving autism care. Pediatrics. 2023;152(5):e2022060584. doi: 10.1542/peds.2022-060584 37795558

[49] Lappé M, Lau L, Dudovitz RN, Nelson BB, Karp EA, Kuo AA. The diagnostic odyssey of autism spectrum disorder. Pediatrics. 2018;141(Suppl 4):S272–9. doi: 10.1542/peds.2016-4300C 29610407 PMC5972532

[50] Malik-Soni N, Shaker A, Luck H, Mullin AE, Wiley RE, Lewis MES, et al. Tackling healthcare access barriers for individuals with autism from diagnosis to adulthood. Pediatr Res. 2022;91(5):1028–35. doi: 10.1038/s41390-021-01465-y 33767375 PMC7993081

[51] Wieckowski AT, Zuckerman KE, Broder-Fingert S, Robins DL. Addressing barriers through tiered diagnostic approaches. Autism Res. 2022;15(12):2216–22. 36254366 10.1002/aur.2832

[52] Thomas KC, Parish SL, Rose RA, Kilany M. Access to care for children with autism in the context of state Medicaid reimbursement. Matern Child Health J. 2012;16(8):1636–44. doi: 10.1007/s10995-011-0862-1 21833759

[53] Leslie DL, Iskandarani K, Dick AW, Mandell DS, Yu H, Velott D, et al. The effects of medicaid home and community-based services waivers on unmet needs among children with autism spectrum disorder. Med Care. 2017;55(1):57–63. doi: 10.1097/MLR.0000000000000621 27547947 PMC5819338

[54] Callaghan T, Sylvester S. Autism spectrum disorder, politics, and the generosity of insurance mandates in the United States. PLoS One. 2019;14(5):e0217064. doi: 10.1371/journal.pone.0217064 31125366 PMC6534322

[55] Davis JM, Harrington MB, Howie FR, Mohammed KS, Gunderson JA. Reducing time to diagnosis of ASD using an integrated community specialty care model. J Pediatr. 2024;270:114009. 38492915 10.1016/j.jpeds.2024.114009

[56] Brinster MI, Brukilacchio BH, Fikki-Urbanovsky A, Shahidullah JD, Ravenscroft S. Improving efficiency and equity in early autism evaluations: The (S)TAAR Model. J Autism Dev Disord. 2023;53(1):275–84. doi: 10.1007/s10803-022-05425-1 35020118 PMC8753324

[57] Habayeb S, Feinberg E, Augustyn M. Primary care-based autism evaluation: reducing wait times while serving predominantly Black and Latinx children insured by Medicaid. Pediatrics. 2025.

[58] McNally Keehn R, Ciccarelli M, Szczepaniak D, Tomlin A, Lock T, Swigonski N. A statewide tiered system for screening and diagnosis of autism spectrum disorder. Pediatrics. 2020;146(2):e20193876. doi: 10.1542/peds.2019-3876 32632023

[59] Hine JF, Allin J, Allman A, Black M, Browning B, Ramsey B, et al. Increasing access to autism spectrum disorder diagnostic consultation in rural and underserved communities: streamlined evaluation within primary care. J Dev Behav Pediatr. 2020;41(1):16–22. doi: 10.1097/DBP.0000000000000727 31490843 PMC6933088

[60] Mazurek MO, Brown K, Curran A. Streamlined referral protocols and diagnostic completion in ASD. J Autism Dev Disord. 2024.

[61] Choi KR, Knight EA, Stein BD, Coleman KJ. Autism insurance mandates in the US: comparison of mandated commercial insurance benefits across states. Matern Child Health J. 2020;24(7):894–900. doi: 10.1007/s10995-020-02950-2 32356129

[62] Barry CL, Epstein AJ, Marcus SC, Kennedy-Hendricks A, Candon MK, Xie M, et al. Effects of state insurance mandates on health care use and spending for autism spectrum disorder. Health Aff (Millwood). 2017;36(10):1754–61. doi: 10.1377/hlthaff.2017.0515 28971920 PMC7011707

[63] Mandell DS, Barry CL, Marcus SC. Effects of autism spectrum disorder insurance mandates on treated prevalence. JAMA Pediatr. 2016;170(9):887–93. 27399053 10.1001/jamapediatrics.2016.1049

[64] Baum RA, Berman BD, Fussell JJ, Patel R, Roizen NJ, Voigt RG, et al. Child health needs and the developmental-behavioral pediatrics workforce supply: 2020-2040. Pediatrics. 2024;153(Suppl 2):e2023063678H. doi: 10.1542/peds.2023-063678H 38300001

[65] Bridgemohan C, Bauer NS, Nielsen BA, DeBattista A, Ruch-Ross HS, Paul LB, et al. A workforce survey on developmental-behavioral pediatrics. Pediatrics. 2018;141(3):e20172164. doi: 10.1542/peds.2017-2164 29453235

[66] McBain RK, Cantor JH, Kofner A, Stein BD, Yu H. State insurance mandates and the workforce for children with autism. Pediatrics. 2020;146(4):e20200836. doi: 10.1542/peds.2020-0836 32900876 PMC7546088

[67] Stavropoulos KK-M, Bolourian Y, Blacher J. A scoping review of telehealth diagnosis of autism spectrum disorder. PLoS One. 2022;17(2):e0263062. doi: 10.1371/journal.pone.0263062 35143494 PMC8830614

[68] Kellom KS, Flaherty CM, Cacia J, et al. Provider and caregiver perspectives on telehealth assessments for autism spectrum disorder. J Dev Behav Pediatr. 2023;44(6):e397–e411. 10.1097/DBP.0000000000001198 37315107

[69] Maenner MJ, Shaw KA, Baio J, et al. Prevalence of ASD among children aged 8 years - ADDM Network, United States, 2016. MMWR Surveill Summ. 2020;69(4):1–12. 10.15585/mmwr.ss6904a1 PMC7119644 32214087

[70] Patrick ME, Williams AR, Shaw KA. Social vulnerability and the prevalence of ASD among 8-year-old children, ADDM Network. Ann Epidemiol. 2025;104:8–14. 40024386 10.1016/j.annepidem.2025.02.014 PMC12004243

[71] Nevison C, Zahorodny W. Race/ethnicity-resolved time trends in US ASD prevalence. J Autism Dev Disord. 2019;49(12):4721–30. 31435818 10.1007/s10803-019-04188-6 PMC6841650

[72] Buck A, Hurewitz S, Franklin MS. Workforce perspective on racial and ethnic equity in early childhood autism evaluation and treatment. Autism. 2024;28(10):2598–611. 38477296 10.1177/13623613241235522

[73] Hotez E, Phan JM, Truong DM. Addressing stigma-related health disparities for autistic individuals through cultural competemility. Curr Psychiatry Rep. 2024;26(12):761–70. 39460907 10.1007/s11920-024-01551-y PMC11706906

[74] Neff J, Knight KR, Satterwhite S. Teaching structure: a qualitative evaluation of a structural competency training. J Gen Intern Med. 2017;32(4):430–3. 27896692 10.1007/s11606-016-3924-7 PMC5377882

[75] Groman R, Ginsburg J, American College of Physicians. Racial and ethnic disparities in health care: a position paper of the American College of Physicians. Ann Intern Med. 2004;141(3):226–32. doi: 10.7326/0003-4819-141-3-200408030-00015 15289223

[76] Majerczyk D, Behnen EM, Weldon DJ, Kanbar R, Hardy YM, Matsuda SK, et al. Racial, ethnic, and sex diversity trends in health professions programs from applicants to graduates. JAMA Netw Open. 2023;6(12):e2347817. doi: 10.1001/jamanetworkopen.2023.47817 38153738 PMC10755626

[77] Snyder JE, Upton RD, Hassett TC, Lee H, Nouri Z, Dill M. Black representation in the primary care physician workforce and its association with population life expectancy and mortality rates in the US. JAMA Netw Open. 2023;6(4):e236687. doi: 10.1001/jamanetworkopen.2023.6687 37058307 PMC10105312

[78] Lee JD, Kang VY, Terol AK, Joo S. Examining the efficacy of culturally responsive interventions for autistic children and their families: a meta-analysis. J Autism Dev Disord. 2025;55(2):706–26. doi: 10.1007/s10803-023-06212-2 38246962 PMC11260274

[79] Attar SM, Benavidez H, Gicheru C, Stahmer AC, Vivanti G. Co-designing a novel service delivery pathway to increase access to autism identification and care. Autism. 2025. 10.1177/13623613251335702 PMC12263326 40346799

[80] Dupras DM, Wieland ML, Halvorsen AJ, Maldonado M, Willett LL, Harris L. Assessment of training in health disparities in US Internal Medicine Residency Programs. JAMA Netw Open. 2020;3(8):e2012757. doi: 10.1001/jamanetworkopen.2020.12757 32777061 PMC7417967

[81] Draper JK, Feltner C, Vander Schaaf EB, Mieses Malchuk A. Preparing medical students to address health disparities through longitudinally integrated social justice curricula: a systematic review. Acad Med. 2022;97(8):1226–35. doi: 10.1097/ACM.0000000000004718 35476779

[82] Castillo EG, Isom J, DeBonis KL, Jordan A, Braslow JT, Rohrbaugh R. Reconsidering systems-based practice: advancing structural competency, health equity, and social responsibility in graduate medical education. Acad Med. 2020;95(12):1817–22. doi: 10.1097/ACM.0000000000003559 32590465 PMC8279228

[83] Hansen H, Metzl JM. New medicine for the U.S. Health Care System: training physicians for structural interventions. Acad Med. 2017;92(3):279–81. doi: 10.1097/ACM.0000000000001542 28079725 PMC5540156

[84] Lai MC, Anagnostou E, Wiznitzer M, Allison C, Baron-Cohen S. Evidence-based support for autistic people across the lifespan. Lancet Neurol. 2020;19(5):434–51. 32142628 10.1016/S1474-4422(20)30034-X

[85] Maye M, Boyd BA, Martinez-Pedraza F. Biases, barriers, and possible solutions. J Autism Dev Disord. 2022;52(9):4206–11. 34529251 10.1007/s10803-021-05250-y PMC8924013

[86] Tafolla M, Lord C. Expanding the autism evidence base. Autism. 2025. 10.1177/13623613251393505 41332364

[87] Fletcher-Watson S, Adams J, Brook K, Charman T, Crane L, Cusack J, et al. Making the future together: Shaping autism research through meaningful participation. Autism. 2019;23(4):943–53. doi: 10.1177/1362361318786721 30095277 PMC6512245

[88] Montiel-Nava C, Navarro C, Abouchard J. Time to listen. J Autism Dev Disord. 2025. 10.1007/s10803-025-06868-y PMC13470970 40372563

[89] Moody EJ, Harris B, Zittleman L, Nease DE, Westfall JM. It’s time for a change!. Cultur Divers Ethnic Minor Psychol. 2019;25(1):113–22. 30714773 10.1037/cdp0000242 PMC6686679

[90] Williams EG, Smith MJ, Boyd B. The role of diversity advisory boards. Autism. 2023;27(3):864–9. 36336998 10.1177/13623613221133633 PMC10073302

[91] Shaia WE, Nichols HM, Dababnah S, Campion K, Garbarino N. Participation of Black families in autism research. J Autism Dev Disord. 2020;50(5):1841–6. 30805765 10.1007/s10803-019-03926-0

[92] Divan G, Bhavnani S, Leadbitter K. Achieving universal health coverage for young children with ASD in LMICs: a review of reviews. J Child Psychol Psychiatry. 2021;62(5):514–35. 33905120 10.1111/jcpp.13404

[93] Grosvenor LP, Croen LA, Lynch FL, et al. Autism diagnosis among US children and adults, 2011-2022. JAMA Netw Open. 2024;7(10):e2442218. 10.1001/jamanetworkopen.2024.42218 PMC11525601 39476234

